# 12.5% of all women's and 7.7% of all men's players from the 2022 and 2023 FIFA World Cups underwent a previous anterior cruciate ligament reconstruction

**DOI:** 10.1002/jeo2.70186

**Published:** 2025-02-24

**Authors:** Diego Costa Astur, Gabriel De Melo Salgado, Marina Dal Piva, David Ken Nagata Radamessi, Edward Patrick Sinibaldi, Antonio Bezerra De Albuquerque Filho, Hassan Ahmad Hauache Neto, Leonardo Adeo Ramos, Moisés Cohen

**Affiliations:** ^1^ Paulista School of Medicine São Paulo São Paulo Brazil; ^2^ Paulista School of Medicine Escola Paulista de Medicina/Universidade Federal de São Paulo São Paulo São Paulo Brazil; ^3^ Cohen Institute, Hospital Israelita Albert Einstein São Paulo São Paulo Brazil

**Keywords:** ACL, anterior cruciate ligament, anterior cruciate ligament revision, football

## Abstract

**Purpose:**

To evaluate the incidence of anterior cruciate ligament (ACL) reconstructions in athletes who participated in the men's 2022 and women's 2023 Federation Internationale de Football Association (FIFA®) World Cups, comparing the incidence between genders, positions and the stages reached by the teams during the competition.

**Methods:**

This is a media analysis study, and data were collected from official club and FIFA® websites: a list of athletes who played during the men's 2022 and women's 2023 FIFA® World Cup, a list of athletes who already did an ACL reconstruction (ACLR) and rehabilitation before the competition, and stages from each team during the competition.

**Results:**

12.5% of the athletes in the women's competition (92 athletes) and 7.7% in the men's competition (64 athletes) underwent ACLR surgery before. When comparing positions (goalkeeper, defence, midfield and forwards), there were no statistical differences between men (*p* = 0.97) and women (*p* = 0.26). According to the competition stage, the prevalence increased from 12.5% in the group stage to 17.4% in the finals (*p* = 0.05) for women and ranged from 6.8% to 7.9% (*p* = 0.87) among men. The number of ACL revision surgeries was 18 in women players (2.4% of total, 19.4% of those operated) and 5 in men players (0.6% of total, 7.5% of those operated). No statistical difference in revision rates between men and women (*p* = 0.16).

**Conclusion:**

In the highest football level competition in the world (FIFA® World Cup), there was no statistical difference between the number of men and women called up and who participated after ACLR, rehabilitation and return to sport (12.5% vs. 7.7%; *p* > 0.05). Furthermore, the incidence of ACL‐operated players belonging to each team that played in the World Cup increased from the stage of groups to the final in the female category (*p* = 0.05), but remained stable in the male category (*p* = 0.87).

**Level of Evidence:**

Not applicable.

**Abbreviations**
ACL: anterior cruciate ligament
ACLR: anterior cruciate ligament reconstruction
FIFA: Federation Internationale de Football Association
IBM: International Business Machine
RTS: return to sports
UEFA: Union of European Football Association

## INTRODUCTION

Football is the most popular sport worldwide. Unfortunately, football‐related knee injuries are common and constitute a serious problem regardless of gender or playing level. The knee injury that probably draws a special attention is the anterior cruciate ligament (ACL) injury. ACL is a major stabiliser of the knee, commonly injured during sports activities, especially those involving knee rotation with the foot fixed on the ground. Its treatment is surgical in the majority of cases and prevents the athlete from practising their sport for a period ranging from 6 to 12 months [9]. But are they playing at a higher level after the ACL reconstruction (ACLR) and rehabilitation programme?

The highest‐level football competition is the Federation Internationale de Football Association (FIFA®) World Cup. Every 4 years, the best national teams from five continents gather to compete in the main tournament of the category, the World Cup. In theory, if an ACL‐reconstructed player is playing in this football competition, it means he is totally recovered from the surgery and rehabilitation process, considering only the best players from each classified country will be selected. Several studies have reported return‐to‐sport rates in athletes undergoing ACLR. Depending on the population investigated, they present mixed results, with return rates to pre‐injury levels varying between 40% and 86% [6, 8, 9, 11]. Evaluating whether there are players with reconstructed ACLs competing in a World Cup is a way of knowing if high‐performance athletes are also able to be among the best after this surgery.

The purpose of this study is to evaluate the incidence of athletes selected and competing in the men's 2022 and women's 2023 FIFA® World Cups who underwent an ACLR previously. The hypothesis is that ACLR is not a limitation for a football player to play in the most important football championship worldwide.

## METHODS

A total of 32 women's teams participated in the 2023 FIFA® Women's World Cup in Australia and New Zealand, with the number of registered players ranging from 23 to 25 per team. Thirty‐two men's teams participated in the 2022 FIFA® World Cup, with the number of registered players ranging from 25 to 26 per team. The inclusion criteria were: players called up and actively participating in the team throughout the tournament, who had undergone at least one ACLR before the tournament, and who returned to play before the tournament began.

It was a media analysis study, and data collection was conducted via the internet, through the official websites of FIFA® (www.fifa.com), the clubs and/or national teams the athletes belong to and confirmed by the time away from activities. Data about registered players from each national team position each one used to play and were registered in these tournaments, as well as match results, are officially available and are public information on the FIFA® website. Information about the previous ACL reconstruction were first analysed by public information on the internet websites and confirmed by the teams the athletes belonged to at the time of the surgery or by their personal staff.

### Statistical analysis

The significance level of the study was 0.05 (5%), and the statistical confidence interval was 95%. The test used to compare the prevalences was the equality of two proportions test by Pearson and ANOVA. In this statistical analysis, the following software was used: SPSS V25 (IBM®) and Excel Office 2017 (Microsoft®).

## RESULTS

**Figure 1:**
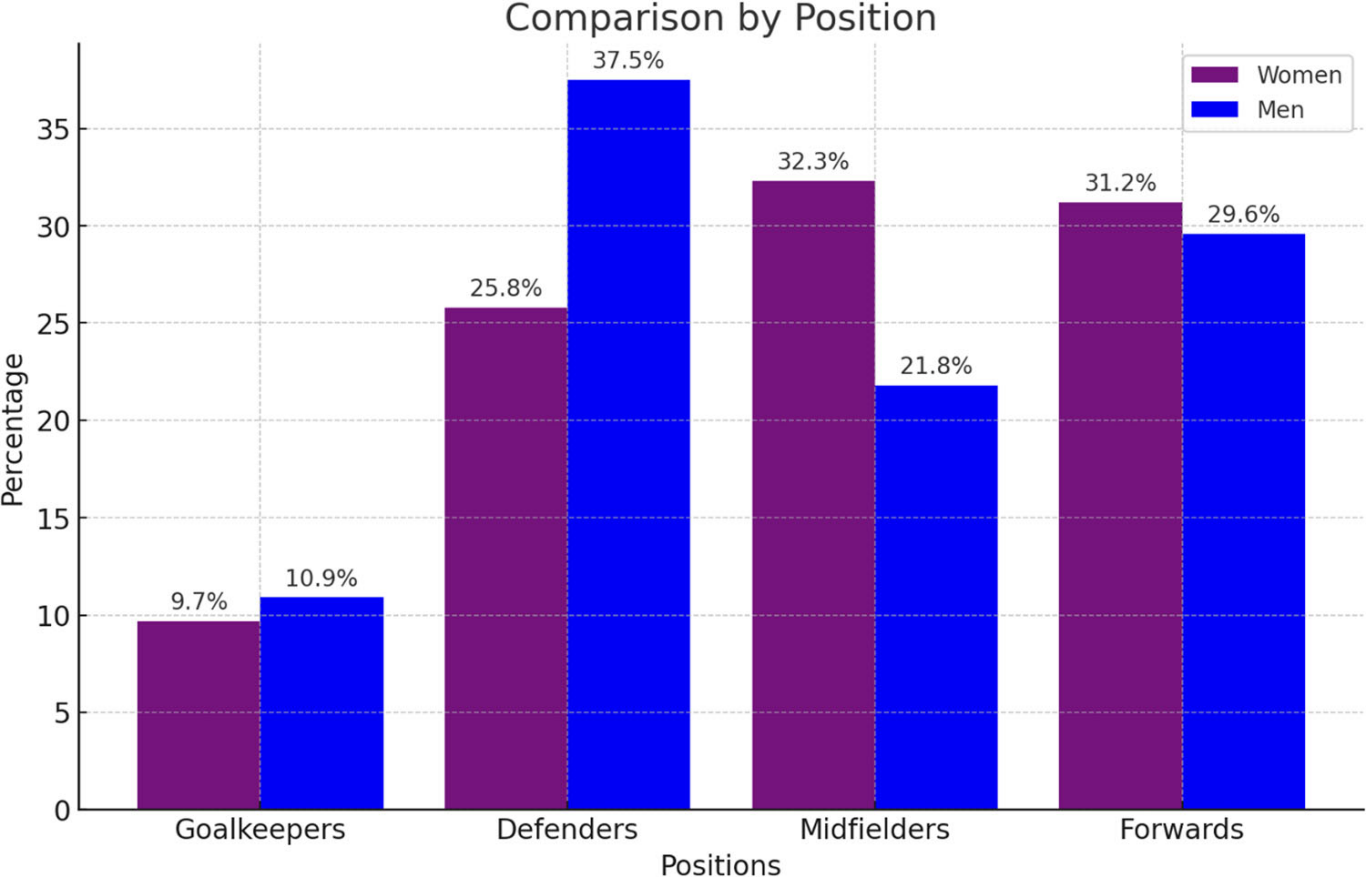
When comparing the prevalence of ACL‐reconstructed players by position, there were no statistical differences between men (*p* = 0.97) and women (*p* = 0.26). When compared by position, there are no differences in the distribution of prevalences (*p* = 0.392). ACL, anterior cruciate ligament.

The total number of athletes who competed in the 2023 and 2022 FIFA World Cups was 738 women and 830 men, respectively. There were 92 ACL‐reconstructed female players (12.5%) and 64 (7.7%) ACL‐reconstructed male players who were rehabilitated in time to compete, with no statistical difference between the groups (*p* = 0.34) (Figure 1).

### Comparison by position

There are no differences in the position men and women played during the World Cup, considering goalkeepers, defenders, midfielders and forwards (*p* > 0.05) (Figure 1).

### Comparison by competition stage

**Figure 2:**
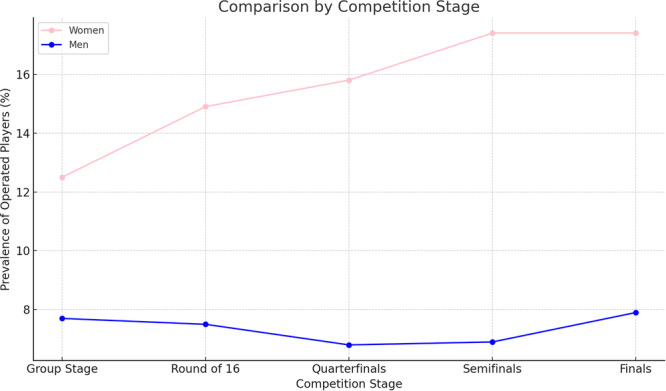
The graph clearly illustrates the upward trend in the prevalence of ACL surgeries among female players as they advance in the competition stages, while the prevalence among male players remains relatively constant: Women increased from 12.5% in the group stage to 17.4% in the finals (*p* = 0.05). Men range from 6.8% to 7.9% (*p* = 0.87). ACL, anterior cruciate ligament.

The prevalence of operated players in the women's group stage was 12.5%. In the round of 16, it was 14.9%; in the quarterfinals, 15.8%; and in the semifinals and finals, 17.4% (*p* = 0.05). The prevalence of operated players in the men's group stage was 7.7%; in the round of 16, it was 7.5%; in the quarterfinals, 6.8%; in the semifinals, 6.9%; and in the finals, 7.9% (*p* = 0.87) (Figure 2).

### ACL revision

**Figure 3:**
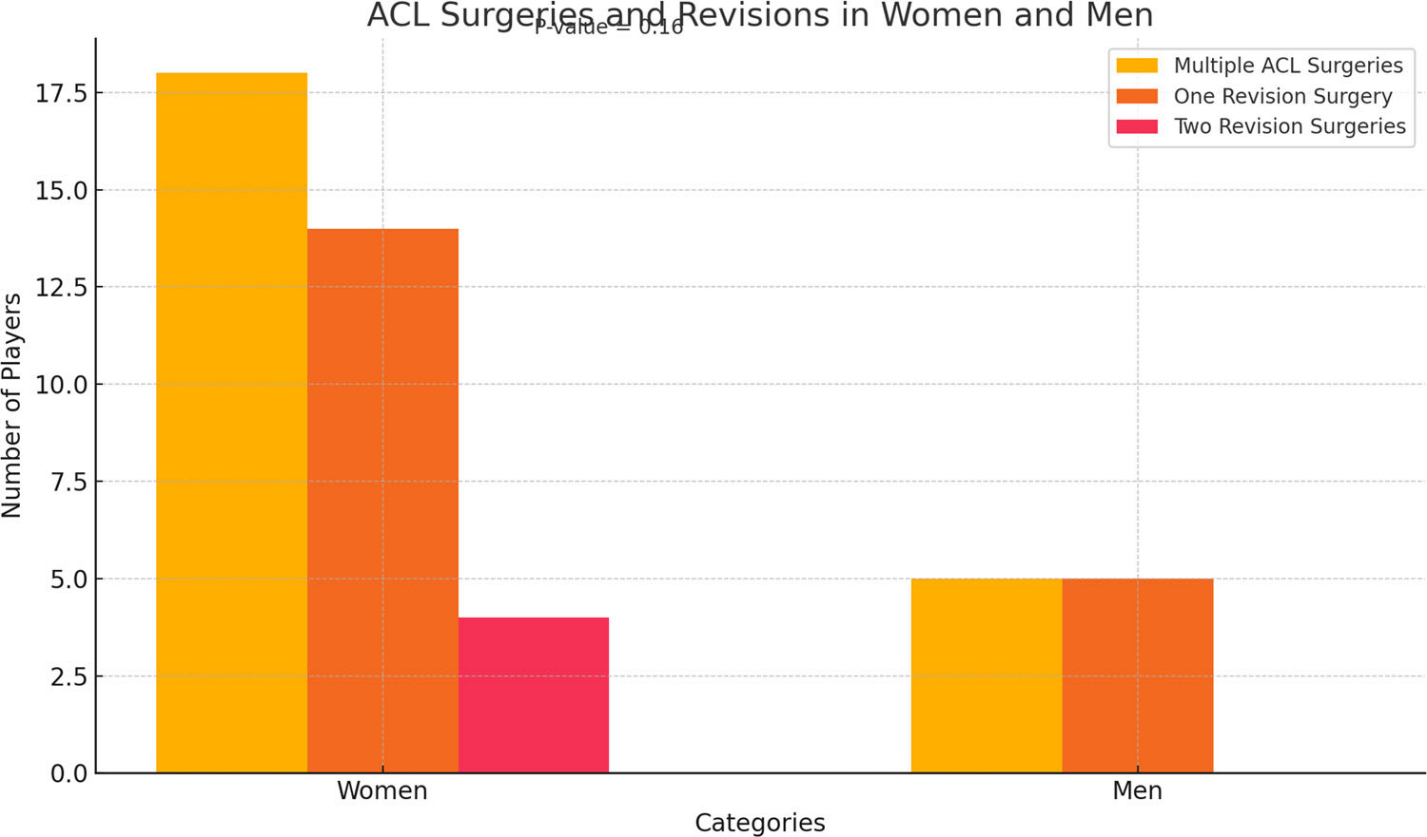
No significant difference in the number of ACL revision surgeries when comparing men and women players (*p *= 0.16), despite 14 women revision cases versus 5 men revision cases. ACL, anterior cruciate ligament.

Eighteen female players with multiple ACL surgeries were presented (2.4% of all players, 19.4% of those operated), 14 of them with one revision surgery, and 4 with two revision surgeries. Five male players had one revision surgery (0.6% of all players, 7.5% of those operated). No statistical difference in revision rates between men and women (*p* = 0.16) (Figure 3).

## DISCUSSION

The most important finding of this study was that some professional football players who underwent an ACLR, after the rehabilitation programme and return to sports (RTS), were able to be called out and participate in the 2022 men's and 2023 women's football World Cup, resulting in 12.5% from all females athletes, and 7.7% of all men athletes during the last tournament. Even that, a successful RTS after ACLR is multifactorial, and dependent on a rehabilitation process [6, 11].

A study with elite players from Union of European Football Association (UEFA) leagues showed that 80% of athletes returned to play after ACLR, with performance equal to or better than pre‐injury levels after three seasons, except for forwards, who showed a continuous decline [3]. Even in teams with the highest level of sports performance, it is possible to find athletes who have undergone this surgery, practicing the sport at a level equal to or even higher than before the injury [12]. In American Major League football, 77% of players returned to the sport after ACLR, and 95% of these returned in the following season. The players' performance was not significantly different from the pre‐injury level [2].

Females have a two to three times increased risk for primary ACL injury compared with males, and they tend to sustain their ACL injury at a younger age [13]. Women are more susceptible to ACL injuries in football due to a combination of anatomical, biomechanical, hormonal, and movement pattern factors [5, 10]. The average ratio in our study among those who returned to play at a high level was 1.61 between F/M, with no significant difference. It means, according to literature datas, there a higher incidence of ACL injury in women, but no difference in the number of player who were able to play during the most important Football competition in the world. Although the number of male football players is still significantly higher than that of female players, women's participation in football has grown substantially in recent decades, with a significant increase in the number of registered female players globally. In 2006, FIFA recorded a total of 265 million football players worldwide, of which 26 million were women, representing about 10% of the total [7].

The prevalence of operated players in the women's World Cup, increased from 12.5% in the group stage to 17.4% in the finals (*p* = 0.05). An explanation for the increase in the incidence of female athletes with ACLR in the final stage of the World Cup may be attributed to the higher prevalence of these injuries among women and the concentration of these injuries in more competitive teams, with higher training loads, which reached the later stages of the competition [4].

ACL re‐rupture in football players is a significant complication with an occurrence rate of approximately 10%. Younger players and men are at higher risk, and proper rehabilitation is crucial for a successful return to the sport [1, 8]. A study of UEFA players found a return‐to‐play rate of 80% after ACLR, with 12% of players experiencing a subsequent re‐rupture. The players' performance generally matched or exceeded that of uninjured controls after three seasons [3]. In the present study, it is interesting that even with two or more surgeries, teams still have a few players in the national teams in this condition, indicating that skills and appropriate rehabilitation programmes are able to improve the capacity to return to play in a high‐level competition.

There are a few limitations in the present study. First, we are evaluating only one type of football championship: the World Cup, which occurs during no more than one month, with no more than seven games, a possible reason to keep an operated player in the team. Second, it is media analysis study, we do not have a direct contact with each athlete, which means, a possible previous surgery in an athlete was lost, although they are public people, and it is difficult not to know the information we need nowadays.

## CONCLUSION

In the highest football level competition in the world (FIFA® World Cup), there was no statistical difference between the number of men and women called up and who participated after ACLR, rehabilitation and RTS (12.5% vs. 7.7%; *p* > 0.05). Furthermore, the incidence of ACL‐operated players belonging to each team that played in the World Cup increased from the stage of groups to the final in the female category (*p* = 0.05), but remained stable in the male category (*p* = 0.87).

## AUTHOR CONTRIBUTIONS

**Diego Costa Astur**: Idea, orientation and revision. **Gabriel De Melo Salgado**: Data collector and writer. **David Ken Nagata Radamessi**: Data collector and writer. **Edward Patrick Sinibaldi**: Data collector and writer. **Antonio Bezerra De Albuquerque Filho**: Data collector. **Hassan Ahmad Hauache Neto**: Data collector. **Moisés Cohen**: Orientation. **Leonardo Adeo Ramos**: Orientation and revision. **Marina Dal Piva**: Data collector.

## CONFLICT OF INTEREST STATEMENT

The authors declare no conflicts of interest.

## ETHICS STATEMENT

The ethics statement is not available.

## Data Availability

All data are freely available on the internet.

## References

[1] Astur DC , Margato GF , Zobiole A , Pires D , Funchal LFZ , Jimenez AE , et al. The incidence of anterior cruciate ligament injury in youth and male soccer athletes: an evaluation of 17,108 players over two consecutive seasons with an age‐based sub‐analysis. Knee Surg Sports Traumatol Arthrosc. 2023;31(7):2556–2562. 36779987 10.1007/s00167-023-07331-0

[2] Erickson BJ , Harris JD , Cvetanovich GL , Bach BR , Bush‐Joseph CA , Abrams GD , et al. Performance and return to sport after anterior cruciate ligament reconstruction in male major league soccer players. Orthop J Sports Med. 2013;1(2):2325967113497189. 26535238 10.1177/2325967113497189 PMC4555483

[3] Forsythe B , Lavoie‐Gagne OZ , Forlenza EM , Diaz CC , Mascarenhas R . Return‐to‐play times and player performance after ACL reconstruction in elite UEFA professional soccer players: a matched‐cohort analysis from 1999 to 2019. Orthop J Sports Med. 2021;9(5):23259671211008892. 34104662 10.1177/23259671211008892 PMC8165856

[4] Geertsema C , Geertsema L , Farooq A , Harøy J , Oester C , Weber A , et al. Injury prevention knowledge, beliefs and strategies in elite female footballers at the FIFA Women's World Cup France 2019. Br J Sports Med. 2021;55(14):801–806. 33397672 10.1136/bjsports-2020-103131

[5] Huston LJ , Greenfield MLVH , Wojtys EM . Anterior cruciate ligament injuries in the female athlete. Potential risk factors. Clin Orthop Relat Res. 2000;372:50–63. 10.1097/00003086-200003000-00007 10738414

[6] Jones M , Hugo Pinheiro V , Balendra G , Borque K , Williams A . No difference in return to play rates between different elite sports after primary autograft ACL reconstruction. Knee Surg Sports Traumatol Arthrosc. 2023;31(12):5924–5931. 37947828 10.1007/s00167-023-07654-y

[7] Kartakoullis NL , Theophanous A . Important parameters of the football industry in Cyprus: challenges and opportunities. Sport J. 2009;12:1–20.

[8] Manara JR , Salmon LJ , Kilani FM , Zelaya de Camino G , Monk C , Sundaraj K , et al. Repeat anterior cruciate ligament injury and return to sport in australian soccer players after anterior cruciate ligament reconstruction with hamstring tendon autograft. Am J Sports Med. 2022;50(13):3533–3543. 36190172 10.1177/03635465221125467

[9] Nitta CT , Baldan AR , Costa LPDB , Cohen M , Pagura JR , Arliani GG . Epidemiology of anterior cruciate ligament injury in soccer players in the Brazilian Championship. Acta Ortop Bras. 2021;29(1):45–48. 33795969 10.1590/1413-785220212901235225 PMC7976862

[10] Rozzi SL , Lephart SM , Gear WS , Fu FH . Knee joint laxity and neuromuscular characteristics of male and female soccer and basketball players. Am J Sports Med. 1999;27(3):312–319. 10352766 10.1177/03635465990270030801

[11] Schiffner E , Latz D , Grassmann JP , Schek A , Thelen S , Windolf J , et al. Anterior cruciate ligament ruptures in German elite soccer players: epidemiology, mechanisms, and return to play. Knee. 2018;25(2):219–225. 29478904 10.1016/j.knee.2018.01.010

[12] Szymski D , Achenbach L , Weber J , Huber L , Memmel C , Kerschbaum M , et al. Reduced performance after return to competition in ACL injuries: an analysis on return to competition in the ‘ACL Registry in German Football’. Knee Surg Sports Traumatol Arthrosc. 2023;31(1):133–141. 35819462 10.1007/s00167-022-07062-8 PMC9859836

[13] Waldén M , Hägglund M , Magnusson H , Ekstrand J . Anterior cruciate ligament injury in elite football: a prospective three‐cohort study. Knee Surg Sports Traumatol Arthrosc. 2011;19(1):11–19. 20532869 10.1007/s00167-010-1170-9

